# Abundant expression of HMGB1 in human T-cell lymphotropic virus type I-infected T-cell lines and high plasma levels of HMGB1 in patients with adult T-cell leukemia

**DOI:** 10.3892/ol.2014.1851

**Published:** 2014-02-03

**Authors:** RYUICHIRO KIMURA, NAOKI MORI

**Affiliations:** 1 Department of Microbiology and Oncology, Graduate School of Medicine, University of the Ryukyus, Nishihara, Okinawa 903-0215, Japan; 2 Transdisciplinary Research Organization for Subtropics and Island Studies, University of the Ryukyus, Nishihara, Okinawa 903-0213, Japan

**Keywords:** HMGB1, adult T-cell leukemia, human T-cell lymphotropic virus type I, Tax

## Abstract

High mobility group box 1 (HMGB1) functions as a chromatin-associated nuclear protein and an extracellular signaling molecule. The concentration of HMGB1 protein and the expression of HMGB1 mRNA were analyzed by ELISA and polymerase chain reaction (PCR), respectively. The present study reports high plasma HMGB1 levels in patients with adult T-cell leukemia [ATL; which is caused by infection with human T-cell lymphotropic virus type I (HTLV-I)] compared with normal controls. In addition, HMGB1 was highly expressed in HTLV-I-infected T-cell lines compared with uninfected T-cell lines. The HTLV-I oncoprotein, Tax, induced extracellular release of HMGB1 in T cells. The results suggest that HMGB1 is a potential biomarker and a therapeutic target for ATL.

## Introduction

Adult T-cell leukemia (ATL) is a highly aggressive malignant disease of CD4^+^ T cells, caused by human T-cell lymphotropic virus type I (HTLV-I) (1). Infection with HTLV-1, the first oncogenic human retrovirus to be identified, also causes various chronic inflammatory disorders, including HTLV-I-associated myelopathy/tropical spastic paraparesis (2). The majority of infected individuals remain clinically asymptomatic, although 2–5% of HTLV-I-infected carriers develop ATL through genetic and epigenetic changes in the cell following a latent period of 40–60 years (3). Tax, the viral oncoprotein, plays a central role in tumorigenesis and contributes to the pathogenesis of ATL and inflammatory diseases by inducing persistent activation of numerous cellular transcription factors, including nuclear factor-κB, cyclic adenosine 3′,5′-monophosphate response element-binding protein and activator protein 1, leading to transactivation of cellular gene promoters (4). However, based on the absence of Tax protein expression in numerous late-stage ATL cells, this viral protein appears to be required only for the initiation of transformation (3,4).

High mobility group box 1 (HMGB1) is an abundant and ubiquitous nuclear protein that binds to DNA and nucleosomes and induces structural changes in the chromatin fiber. It regulates numerous cellular activities, including transcription, replication and repair (5). In addition, HMGB1 is released from damaged, necrotic or activated immune cells and functions as an extracellular signaling molecule (6,7). Thus, intranuclear and extranuclear HMGB1 are therapeutic targets for inflammation and infection (8). Previous studies have identified the roles of HMGB1 in cancer (9), with high protein expression in colon, breast, lung, prostate, cervical and gastric cancer, hepatocellular carcinoma and leukemia compared with normal tissues and healthy controls (10).

More recently, Zhang *et al* (11) have demonstrated that HMGB1 expression is upregulated by HTLV-I Tax in T cells. However, the expression levels of HMGB1 in HTLV-I-infected T-cell lines have not been determined. In the present study, the levels HMGB1 in several T-cell lines and in the plasma of patients with ATL were analyzed.

## Materials and methods

### Cell lines

The HTLV-I-infected T-cell lines, MT-2 (12), MT-4 (13), C5/MJ (14), SLB-1 (15), HuT-102 (16), MT-1 (17), TL-OmI (18) and ED-40515(−) (19), were cultured in RPMI-1640 medium supplemented with 10% heat-inactivated fetal bovine serum. MT-2, MT-4, C5/MJ and SLB-1 are HTLV-I-transformed T-cell lines established by an *in vitro* coculture protocol. MT-1, TL-OmI and ED-40515(−) are T-cell lines of leukemic cell origin established from patients with ATL. HuT-102 was also established from a patient with ATL and constitutively expresses viral genes, but its clonal origin is not clear. In JPX-9 cells, Tax is under the transcriptional control of the metallothionein gene promoter and can be induced by the addition of CdCl_2_ to the medium (20). Experiments using JPX-9 cells were carried out after a 24-h cultivation period in the absence and presence of 20 *μ*M CdCl_2_.

### Patients and plasma samples

The diagnosis of acute ATL was based on clinical features, hematological findings and the presence of anti-HTLV-I antibodies in the serum. Plasma samples were collected at the time of admission to the Naha City Hospital (Naha, Japan) prior to chemotherapy and stored at −80°C until use. Informed consent was obtained from all blood donors.

### Polymerase chain reaction (PCR)

Total RNA was prepared from various cell cultures using TRIzol (Invitrogen Life Technologies, Carlsbad, CA, USA) according to the instructions provided by the manufacturer. First-strand cDNA was synthesized from 1 *μ*g total cellular RNA using a PrimeScript RT-PCR kit (Takara Bio Inc., Otsu, Japan) with random primers. The primer sequences for HMGB1, receptor for advanced glycation end products (RAGE), Tax and GAPDH are listed in Table I. PCR was halted during the exponential phase of DNA amplification and the reaction products were fractionated on 2% agarose gels and visualized by ethidium bromide staining. The obtained bands of amplified DNA were quantified using Image J (National Institutes of Health, Bethesda, MD, USA).

### Assay for HMGB1

The concentrations of HMGB1 were measured in cultured supernatants from JPX-9 cells and plasma samples from patients and healthy donors using a commercially available ELISA kit II (Shino-Test Corporation, Tokyo, Japan) according to the manufacturer’s instructions. The minimum detection level for HMGB1 was 1 ng/ml.

### Statistical analysis

Differences between groups were examined for statistical significance using the unpaired Student’s t-test. P<0.05 was considered to indicate a statistically significant difference.

## Results

### Expression of HMGB1 and RAGE mRNA in HTLV-I-infected T-cell lines

**Figure 1:**
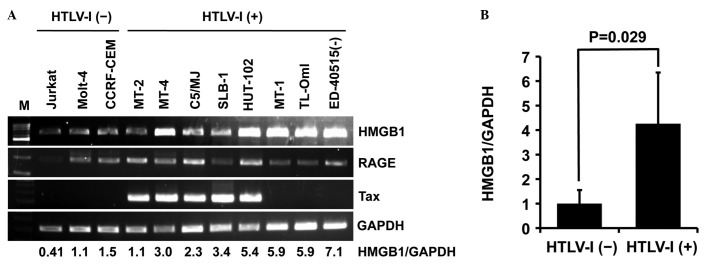
(A) Polymerase chain reaction analysis of HMGB1, RAGE, Tax and GAPDH mRNA expression in HTLV-I-infected T-cell lines. The HMGB1/GAPDH ratio was calculated by densitometric analysis of the bands. (B) Comparison of HMGB1 mRNA expression levels between HTLV-I-infected T-cell lines and uninfected T-cell lines. Data are presented as mean ± standard deviation. HMGB1, high mobility group box 1; RAGE, receptor for advanced glycation end products; HTLV, human T-cell lymphotropic virus.

First, the expression levels of HMGB1, RAGE (HMGB1 receptor) and Tax were analyzed by PCR in eight HTLV-I-infected T-cell lines [MT-2, MT-4, C5/MJ, SLB-1, HuT-102, MT-1, TL-OmI and ED-40515(−)]. HTLV-I-transformed T-cell lines (MT-2, MT-4, C5/MJ and SLB-1) and HuT-102 constitutively expressed Tax mRNA, but ATL-derived T-cell lines [MT-1, TL-OmI and ED-40515(−)] did not (Fig. 1A). PCR also revealed high expression of HMGB1 mRNA in all HTLV-I-infected T-cell lines except MT-2 compared with uninfected T-cell lines (Jurkat, Molt-4 and CCRF-CEM), regardless of Tax expression (Fig. 1A). The relative expression of HMGB1 in HTLV-I-infected T-cell lines was four-fold higher than in uninfected T-cell lines (Fig. 1B). By contrast, RAGE was expressed in all T-cell lines except Jurkat, regardless of HTLV-I infection, although its expression levels varied widely (Fig. 1A).

### Tax protein enhances HMGB1 release in T cells

**Figure 2:**
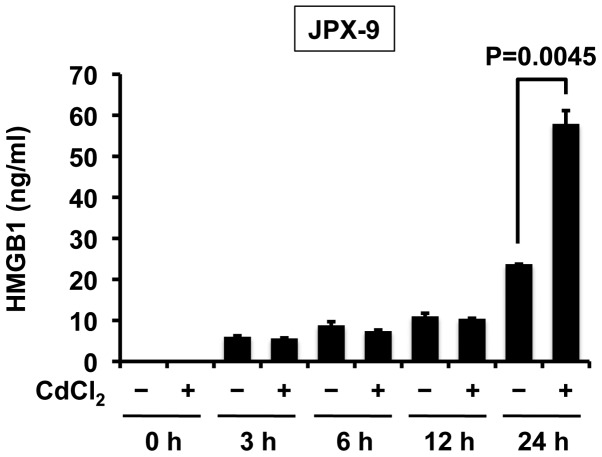
Tax enhances release of HMGB1 in T cells. JPX-9 cells were treated with or without 20 *μ*M CdCl_2_ for the indicated time periods and the culture supernatants were harvested. HMGB1 protein concentrations in cultured supernatants from JPX-9 cells were measured by ELISA. Data are presented as mean ± standard deviation. HMGB1, high mobility group box 1.

Next, the study sought to discern whether Tax induces the release of HMGB1 in T cells. Treatment of JPX-9 cells (a Jurkat subline that carries the Tax gene under the control of the metallothionein gene promoter) with CdCl_2_ rapidly induced Tax expression (20). ELISA revealed that Tax enhanced the release of HMGB1 in T cells in the 24 h after treatment with CdCl_2_ (Fig. 2).

### Plasma levels of HMGB1 in patients with ATL

**Figure 3:**
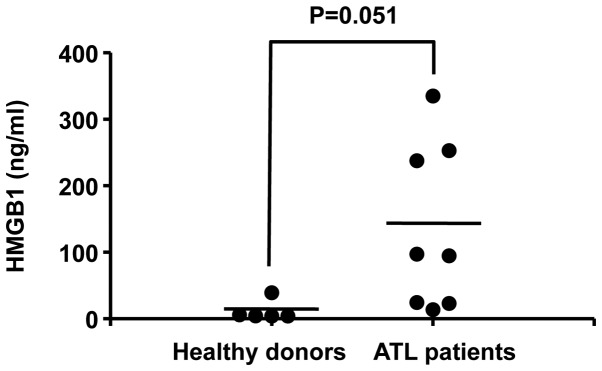
HMGB1 release is high in the plasma of ATL patients. Plasma HMGB1 levels in healthy donors (n=5) and patients with acute ATL (n=8). Horizontal lines represent mean values. HMGB1 protein concentrations in plasma samples were measured using an ELISA. HMGB1, high mobility group box 1; ATL, adult T-cell leukemia.

Finally, HMGB1 levels in the plasma of ATL patients were investigated. The plasma levels of HMGB1 in patients with acute ATL (n=8) tended to be higher than those in healthy donors (n=5), albeit without statistical significance (P=0.051) (Fig. 3). The mean plasma HMGB1 levels in ATL patients (134 ng/ml) were ten-fold higher than those of healthy donors (11 ng/ml). These data demonstrate that HMGB1 is markedly released in the plasma of ATL patients.

## Discussion

The HTLV-I transactivator protein, Tax, is a protein of significant interest in HTLV-I pathogenesis, as it is a potent activator of a variety of transcription pathways and is in itself sufficient to immortalize T cells *in vitro.* Thus, it plays an important role in cellular transformation (3,4). Recently, it has been reported that Tax is involved in upregulation of HMGB1 expression by interaction with CCAAT/enhancer binding protein (11). The present study found that HMGB1 mRNA was abundantly expressed in HTLV-I-infected T-cell lines. In addition, Tax increased HMGB1 secretion in T cells. These results are consistent with those reported previously (11). Tax expression did not correlate with the upregulation of HMGB1 mRNA in HTLV-I-infected T-cell lines. However, significantly higher plasma levels of HMGB1 were noted in ATL patients with peripheral leukemic cells negative for Tax protein than in healthy donors. Thus, it may be that another factor is essential for the induction of HMGB1 expression and secretion in ATL.

While the exact function of HMGB1 protein is not clear at present, it is reported to play important roles in sustained angiogenesis, evasion of apoptosis, growth signal self-sufficiency, insensitivity to antigrowth signals, the inflammatory microenvironment, immortalization, tissue invasion and metastasis in cancer cells (10). In addition, HMGB1 is reported to promote drug resistance in osteosarcoma (21). Previous studies have also shown that endogenous HMGB1 regulates autophagy (22) and that HMGB1-induced autophagy promotes resistance to chemotherapy in leukemia cells (23). Therefore, HMGB1 appears to play important roles in malignant progression, inflammation, organ infiltration and drug resistance in HTLV-I-associated diseases, including ATL. These findings suggest that HMGB1 is a potential biomarker and therapeutic target for ATL.

In conclusion, the present study demonstrated that there are elevated plasma HMGB1 levels in patients with ATL and an abundant HMGB1 expression in HTLV-I-infected T-cell lines.

## Figures and Tables

**Table I:** Primer sequences.

Gene name	Forward sequence	Reverse sequence
HMGB1	5′-ATGGGCAAAGGAGATCCTAAGAA-3′	5′-TTATTCATCATCATCATCTTCTT-3′
RAGE	5′-ATGGAAACTGAACACAGGCC-3′	5′-CACACATGTCCCCACCTTAT-3′
Tax	5′-CCGGCGCTGCTCTCATCCCGGT-3′	5′-GGCCGAACATAGTCCCCCAGAG-3′
GAPDH	5′-GCCAAGGTCATCCATGACAACTTTGG-3′	5′-GCCTGCTTCACCACCTTCTTGATGTC-3′

HMGB1, high mobility group box 1; RAGE, receptor for advanced glycation end products.
